# Evaluation of Anterior Segment Changes of Patients Taking Alpha1-Blockers by Ultrasound Biomicroscopy in the Drug-free Period

**DOI:** 10.4274/tjo.45336

**Published:** 2017-08-15

**Authors:** Yeliz Acar, Kadir Eltutar, Sibel Zırtıloğlu

**Affiliations:** 1 İstanbul Training and Research Hospital, Ophthalmology Clinic, İstanbul, Turkey

**Keywords:** Alpha1 blocker, Anterior segment, ultrasound biomicroscopy

## Abstract

**Objectives:**

To evaluate and compare anterior segment changes in patients taking alpha-1 (α1) blockers (tamsulosin, terazosin, doxazosin, alfuzosin) for benign prostatic hypertrophy, during drug intake and drug-free period, using ultrasound biomicroscopy (UBM).

**Materials and Methods:**

In this prospective study, UBM was done before and after pupil dilatation in 31 phakic eyes of 19 male patients taking α1-blockers. Undilated and dilated UBM was repeated before cataract extraction, after stopping the drug for 10 days. On ideal images, pupil diameter (PD), anterior chamber depth (ACD), anterior chamber angle (ACA), and angle opening distances at points 500 µm and 250 µm from the scleral spur (AOD500 and AOD250) values were noted and changes in parameters were evaluated to reveal any changes that occurred after discontinuing the drug. No patient in the study was previously or currently using any other α1-adrenergic antagonist medication. Exclusion criteria for all patients included a history of diabetes mellitus, systemic hypertension, glaucoma, pseudoexfoliation syndrome, chronic use of medicated eye drops, and previous ocular surgery.

**Results:**

PD, ACD, ACA, AOD500 and AOD250 values measured before pupil dilatation in the drug-free period were not significantly different from those measured during α-blocker intake (p>0.05). In dilated eyes, the mean value of AOD500 was 0.35±0.08 mm during drug usage and 0.39±0.08 mm in the drug-free period. The mean value of AOD250 was 0.23±0.06 mm during drug usage and 0.26±0.07 mm after discontinuation. These increments were statistically significant (p<0.05, z=-3.699, z=-2.984). On the other hand, there were no significant differences in ACD, ACA, or PD values in dilated eyes after discontinuing α1-blockers (p>0.05).

**Conclusion:**

The interruption of taking α1-blockers in patients who have benign prostatic hypertrophy does not seem to influence anterior segment parameters generally. However, further investigation is needed.

## INTRODUCTION

Intraoperative floppy-iris syndrome (IFIS) associated with the usage of tamsulosin (Flomax®), first described by Chang and Campbell^1^ in 2005, is defined by iris billowing, prolapse, and progressive pupil constriction during cataract surgery. Since then, several reports have confirmed IFIS and its relationship with tamsulosin. There is a wide spectrum of clinical expression of this syndrome, with some patients showing signs in one eye only or having asymmetric involvement between fellow eyes.^2,3^ In addition, there are even several reports of patients developing IFIS for a long time after discontinuing tamsulosin.^1,4^

The pathophysiology of IFIS is not well described. Tamsulosin is an α1-adrenergic antagonist which is used to treat urinary retention from benign prostatic hypertrophy. Because of this, it is prescribed by urologists to block α1-receptors on the smooth muscle of the prostate, leading to muscle relaxation and relief of bladder outflow obstruction. Alpha1-adrenergic receptors are also found on the iris dilator muscle.^5,6^ Recent studies suggest that iris dilator muscle thickness is reduced in individuals with a history of tamsulosin use.^7,8^ It has been claimed that blocking the α1-adrenergic receptors on the iris dilator muscle leads to disuse atrophy, deficient mydriasis, and irregular iris behavior during intraocular surgery.

It is known that the risk of IFIS cannot be eliminated, but it can be reduced by discontinuing α-blockers. The biological half-life of tamsulosin is 48-72 hours.^9^ Therefore, discontinuing the drug 4-7 days before surgery may be beneficial, but not able to completely prevent IFIS. There is no relationship between the duration of tamsulosin intake and IFIS.1 IFIS associated with tamsulosin and other α-adrenoreceptor blockers seems to be a partially permanent pathology. Although pupil dilation improves and iris billowing decreases when tamsulosin is discontinued 1-2 weeks before cataract surgery, the risk of IFIS persists up to a year after discontinuation.^1,2^ The current prospective study was designed to determine whether there are any preoperative changes in anterior segment (AS) parameters measurable by ultrasound biomicroscopy (UBM) after discontinuing α1-blocker.

## MATERIALS AND METHODS

Approval for the study was obtained from the Ethics Review Committee of the İstanbul Training and Research Hospital (protocol number: 473). All research protocols adhered to the tenets of the Declaration of Helsinki and all volunteers went through a complete informed consent process. Patients were chosen from the list of those scheduled to have cataract surgery in the Ophthalmology Department of the İstanbul Training and Research Hospital in İstanbul, Turkey.

Phakic eyes of male patients with current use of tamsulosin (Flomax®, Boehringer Ingelheim), terazosin (Hytrin®, Abbott), doxazosin (Cardura®, Pfizer), or alfuzosin (Xatral®, Sanofi Aventis) were included in the study. No patient in the study was previously or currently using any other α1-adrenergic antagonist medication. Exclusion criteria for all patients included a history of diabetes mellitus, systemic hypertension, glaucoma, pseudoexfoliation syndrome, chronic use of medicated eyedrops (antiglaucomatous, steroids, non-steroidal anti-inflammatory drugs, antibiotics, etc.), and previous ocular surgery.

Patients using a α1 antagonist and scheduled for cataract surgery in our clinic underwent UBM (Sonomed-VuMax II®) both immediately before and 30 min after pupil dilatation with 2.5% phenylephrine (Mydfrin®, Alcon) and 1% tropicamide (Tropamid®, Bilim). Then they were requested to stop using the α1-blocker for 10 days. UBM was repeated on the day of surgery, both before and after pupil dilatation. Pupil diameter (PD), anterior chamber depth (ACD), anterior chamber angle (ACA), angle opening distances (AOD) at both 500 µm and 250 µm (AOD250) were measured from the UBM scans and evaluated to determine whether any significant changes occurred in the AS in the drug-free period.

All measurements were taken with the patient in supine position with dim ambient lighting to provide natural pupil dilation. Topical 0.5% propacaine HCl (Alcaine®, Alcon) was instilled before the procedure. For scanning, a silicone cup of the appropriate size (18, 20 or 22 mm) was gently placed between the superior and inferior fornices. Patients were instructed to keep their eyes open and look at a fixed point on the ceiling, sufficient saline was put in the cup for immersion, and the scan was initiated. Firstly, axial images of the AS were taken, radial section images of the angle on superior, inferior, lateral and medial quadrants were taken instantly. For the ideal images and to take consistent measurements, we took care to ensure axial and vertical alignment. While taking the axial section images, the probe was placed vertically to the limbus to get the best reflectance of iris. We also ensured the visibility of the scleral spur on all images.

Both the scans and measurements were taken by the same observer. All measurements were taken at least twice at different times by the same individual. PD, ACD, ACA, AOD500, and AOD250 were measured using the scales on the device software, consistent with the method suggested by Pavlin et al.^10^

(Figure 1, 2, 3).

### Statistical Analysis

Mean and standard deviation were used in descriptive statistical analyses. Wilcoxon signed-rank test was used to analyze the repeated measurements in related groups at different times. Statistical Package for the Social Sciences (SPSS) version 20.0 (IBM Corporation, USA) software was used for all analyses.

## RESULTS

Thirty-one phakic eyes of 19 male patients were included in the study. The mean age of patients was 71.3±0.7 years. Pupil diameters were analyzed first. The mean values of all other parameters and details are shown in Table 1. The mean non-dilated PD in the drug-free period was 3.45±0.72 mm. There was no difference in this value during α1-blocker use. The other parameters also did not differ based on drug use in non-dilated eyes (p>0.05) (Table 2).

There were no significant differences in dilated PD, ACD, or ACA measurements after discontinuing α1 antagonists (p>0.05). However, the mean values of AOD500 and AOD250 with dilated pupils were statistically higher in the drug-free period. The mean value of AOD250 was 0.24±0.06 mm during α-blocker use and 0.26±0.07 mm in the drug-free period, and these values for AOD500 were 0.36±0.08 mm and 0.39±0.08 mm, respectively. The differences were statistically significant (p<0.05, z=-3.699, z=-2.984). Other measurements and statistical data are shown in Table 3.

## DISCUSSION

Since the first report of IFIS in 2005,^1^ there has been a great deal of interest in better understanding this condition in order to ensure the safety of cataract surgery in patients taking tamsulosin. The pathophysiology of IFIS is not well understood, and current research is mostly focused on the direct effect of α1-adrenergic antagonists on the iris dilator muscle. Chronic receptor blockade could lead to iris vascular dysregulation, subsequent secondary atrophy of the iris dilator muscle, and finally, the anomalous iris behavior seen in IFIS. A recent prospective study has shown that not only α-blocker intake but benzodiazepines, quetiapine, and finasteride were all independently associated with IFIS.^11^ Furthermore, Matsuo et al.^12^ reported that they observed IFIS in 3 cases with a long-term history of using antipsychotic drugs such as haloperidol, risperidone, olanzapine, chlorpromazine, quetiapine, aripiprazol, without a history of using selective α1-blocker.

Chang and Campbell^1^ construed that because of the long half-life of tamsulosin (48-72 hours), the iris dilator muscle became atrophic and this led to IFIS. But in other studies which investigated drug accumulation in melanocytes, while the accumulation of drugs like levofloxacin and chloroquine was shown in iris melanocytes, α1-blockers were not involved.^13^ Goseki et al.^13^ demonstrated that bunazosin, which is a selective α1 antagonist, accumulated in melanocytes and they proposed that this accumulation might lead to IFIS, as Chang et al.^4^ stated.

The half-life of tamsulosin is 48-72 hours.^9^ Therefore, it is believed that discontinuing the drug 4-7 days before the surgery may be beneficial. We wondered if there are any changes in AS parameters after discontinuing α-blocker for 10 days. Therefore, we decided to investigate this in the eyes of our cataract patients prior to surgery. We used UBM to evaluate AS parameters in this study in order to determine whether there were any differences in eyes when the patients stopped taking α-1 antagonists.

Shtein et al.^14^ used AS optical coherence tomography (AS-OCT) to assess iris morphology, including iris thickness and PD in patients using tamsulosin. Although some other studies using AS-OCT describe changes in iris thickness associated with tamsulosin use in patients with glaucoma,^8^ similar to Tufan et al.,^15^ they did not detect differences in iris thickness in their study. In contrast to the differences in pupil size seen by Tufan et al.^15^ on AS-OCT, Shtein et al.^14^ found that photopic pupil measurements on AS-OCT were not significantly different between patients who had taken tamsulosin and those who had not. Their clinical measurements of pharmacologically dilated pupil size before surgery found significantly smaller pupils in patients who had used tamsulosin than in control patients, in contrast to the findings in a study by Cooney et al.^16^ They believed that their study used stronger pharmacologic pupil dilators because patients were being prepared for surgery rather than being dilated in the clinic. However, even in this study, preoperative pupil size was not directly associated with clinical manifestations of IFIS and thus provided no information that was predictive of intraoperative iris behavior.

Tufan et al.^15^ found that scotopic PD was similar in patients using α-blocker and those who had never used an α-blocker (3.99±1.11 vs. 3.74±1.35, nonsignificant). They noted a significantly reduced photopic PD (2.89±0.55 vs. 3.62±0.64, p<0.001) and an increased scotopic/photopic PD (1.42±0.44 vs. 1.02±0.30, p<0.001) in the study group and concluded that evaluating changes in PD might be more useful for predicting IFIS than evaluating iris structural alterations.

Yuksel et al.^17^ also investigated whether there were any differences in AS parameters between three patient groups: treated with tamsulosin, treated with doxazosin, and untreated. They used Pentacam to examine the patients in standard dim light conditions. They found that PD, ACD, and ACA were decreased in the first two groups, while central corneal thickness and corneal volume were similar in all groups.

In our study we did not use AS-OCT or Pentacam, but ultrasound biomicroscopy, which is more subjective, so we ensured that all examinations and measurements were performed by the same physician under the same room light conditions. Unexpectedly, we did not find any significant differences after the patients stopped taking α1-blockers for 10 days. Including pupil diameters, none of the parameters except AOD250 and AOD500 changed after discontinuing the drug. However, the difference appeared only in dilated eyes. Perhaps AOD measurements increased after discontinuing in dilated eyes because of the effect of α1 antagonists on the pupil dilatation mechanism. But in contrast, there were no differences in pupil diameters. Our patients were dilated just before the cataract extraction, so strong pharmacological dilatation might cause that. There were, of course, significant increases in pupil diameters in some individuals after interruption of α1-blocker usage, but this variation in pupil diameters may be related to the duration of drug intake.

### Study Limitations

We could not find any other studies that investigated differences after discontinuing α-blockers. Therefore, more prospective studies using more objective AS imaging techniques are needed to compare our results. We used ultrasound biomicroscopy, which is subjective enough to affect the final measurements, so we took care that all measurements were done by the same physician under the same room light conditions. Another limitation was the insufficient number of patients included in the study.

## CONCLUSION

After stopping α1-blockers intake, there were no significant differences in AS parameters measured before pupil dilatation. Furthermore, of the measurements taken after pupil dilatation, only angle opening distances were increased after discontinuing the drugs.

## Figures and Tables

**Table 1:**
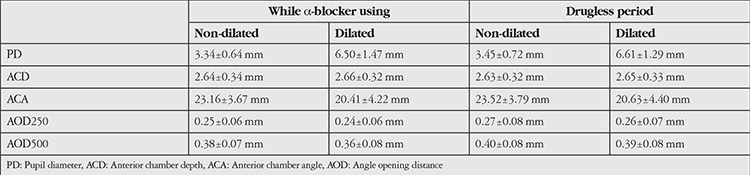
The mean values of anterior segment parameters

**Table 2:**
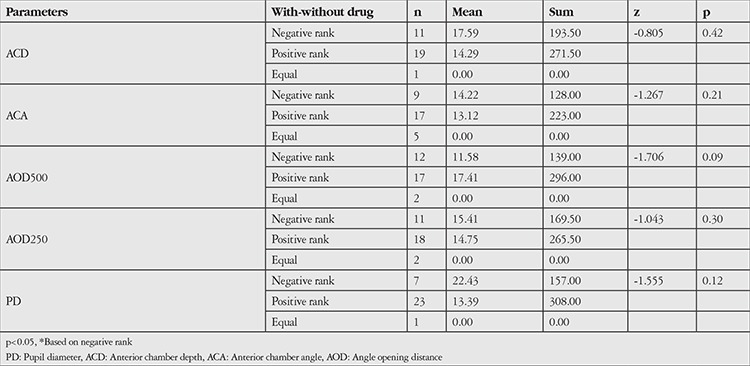
Anterior segment parameters Wilcoxon Signed-Rank test results (non-dilated)

**Table 3:**
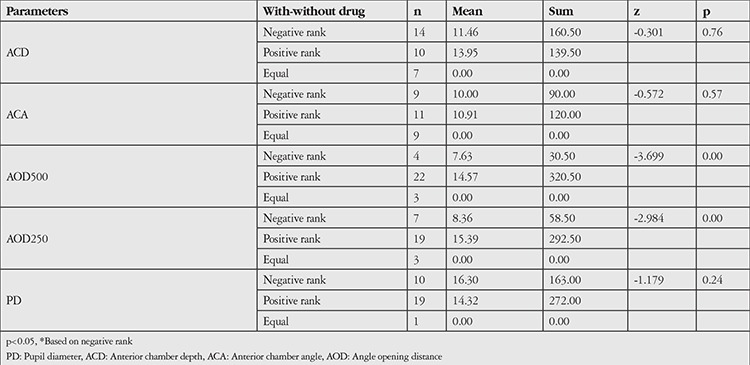
Anterior segment parameters Wilcoxon Signed-Rank test results (dilated)

**Figure 1:**
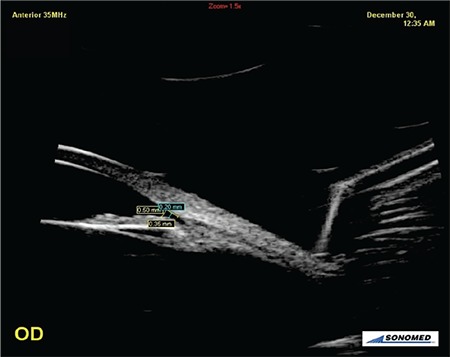
Angle opening distance (AOD) 500 and AOD250 measurements after pupil dilatation during alpha1 blocker usage (AOD500-250: AOD at points 500 µm and 250 µm away from scleral spur) OD: Right eye

**Figure 2:**
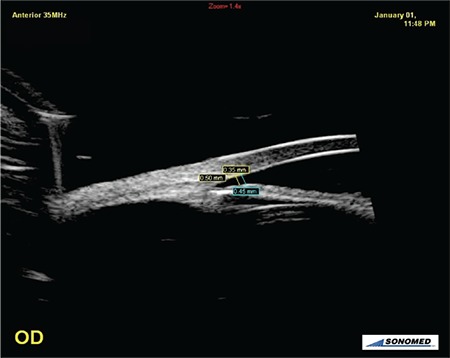
Angle opening distance (AOD) 500 and AOD250 measurements before dilatation after discontinuing alpha1 blocker (AOD500-250: AOD at points 500µm and 250µm away from scleral spur) OD: Right eye

**Figure 3:**
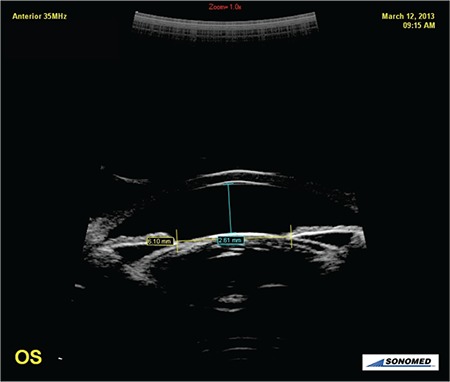
Anterior chamber depth and pupil diameter measurements after pupil dilatation during alpha1 blocker usage OS: Left eye
